# *Rhodiola crenulata* extract regulates hepatic glycogen and lipid metabolism *via* activation of the AMPK pathway

**DOI:** 10.1186/s12906-016-1108-y

**Published:** 2016-05-17

**Authors:** Kuen-Tze Lin, Shih-Wei Hsu, Feng-Yi Lai, Tsu-Chung Chang, Li-Shian Shi, Shih-Yu Lee

**Affiliations:** Graduate Institute of Medical Sciences, National Defense Medical Center, Taipei, Taiwan; Department of Radiation Oncology, Tri-Service General Hospital, National Defense Medical Center, Taipei, Taiwan; Department of Neurosurgery, Taichung Armed Forces General Hospital, Taichung, Taiwan; Graduate Institute of Aerospace and Undersea Medicine, National Defense Medical Center, Taipei, Taiwan; Department of Biochemistry, National Defense Medical Center, Taipei, Taiwan; Department of Biotechnology, National Formosa University, Yunlin, Taiwan

**Keywords:** *Rhodiola crenulata*, Lipogenesis, Glycogen synthesis, AMPK

## Abstract

**Background:**

Metabolic syndrome may lead to many complications, such as nonalcoholic fatty liver disease (NAFLD). A natural and effective therapeutic agent for patients with NAFLD is urgently needed. In a previous study, we showed that *Rhodiola crenulata* root extract (RCE) regulated hepatic gluconeogenesis through activation of AMPK signaling. However, the manner in which RCE regulates hepatic lipid and glycogen metabolism remains unclear. The current study was conducted to investigate the effects of RCE on hepatic glycogen and lipid metabolism, as well as the mechanisms underlying such effects.

**Methods:**

Human hepatoma HepG2 cells were treated with RCE for 6 h under high glucose conditions, after which glycogen synthesis, lipogenesis, and relative gene expression were examined. In addition, lipogenesis-related genes were investigated in vivo.

**Results:**

RCE significantly increased glycogen synthesis and inhibited lipogenesis, while regulating genes related to these processes, including glycogen synthase kinase 3β (GSK3β), glycogen synthase (GS), fatty acid synthase (FAS), CCAAT/enhancer-binding protein (C/EBP), and sterol regulatory element-binding protein 1c (SREBP-1c). However, the effects caused by RCE were neutralized by compound C, an AMPK antagonist. Further studies showed that expression levels of lipogenic genes decreased at the protein and mRNA levels in the rat liver.

**Conclusions:**

Our results demonstrate that RCE regulates hepatic glycogen and lipid metabolism through the AMPK signaling pathway. These results suggest that RCE is a potential intervention for patients with NAFLD.

## Background

Modern lifestyles have resulted in a sharp increase in the occurrence of metabolic syndrome in recent decades. Metabolic syndrome is primarily caused by insulin resistance and associated with obesity and central fat distribution [1]. In addition, metabolic disorder may subsequently develop into cardiovascular disease and type II diabetes mellitus [2]. Thus, it has become a major public health concern in developed nations [3]. Insulin resistance contributes to excessive concentrations of free glucose and fatty acids in the circulatory system, which may cause increased oxidative stress, release of inflammatory cytokines, impaired β-oxidation of free fatty acids, and abnormal metabolism [4]. Among the effects of insulin resistance, abnormal hepatic glucose and lipid metabolism are the major causes of metabolic syndrome. One of the consequences of insulin resistance is redirection of excess glucose to de novo lipogenesis instead of glycogen synthesis in the liver [5]. Increased de novo lipogenesis leads to fat accumulation in the liver and is linked to the pathological progression of cirrhosis, nonalcoholic fatty liver disease (NAFLD), hepatitis, and liver cancer [6]. Thus, reduction of excessive hepatic lipidosis is an urgent concern for patients with metabolic syndrome.

Enhanced lipogenesis and decreased glycogen synthesis are hallmarks of hepatic insulin-resistance, which might subsequently lead to the development of type II diabetes mellitus [7]. The liver is the primary organ responsible for glycogen and lipid metabolism. Biosynthesis of glycogen and lipids is the primary means by which the body stores excess nutrients and is strictly controlled by a complex network of hormones and metabolic signals. Under normal conditions, glycogen is the primary storage form of excess energy. Glycogen production is regulated primarily *via* enzymes critically involved in glycogen metabolism, including glycogen synthase kinase 3β (GSK3β) and glycogen synthase (GS) [8]. However, insulin resistance shifts the major form of energy storage from glycogen to triglycerides (TG) in the liver [9], consistent with reduced GS activity in patients with type II diabetes [10]. These shifts in energy storage mechanisms increase lipogenesis, enhance cholesterol synthesis, and decrease fatty acid β-oxidation; eventually, such effects may lead to lipotoxicity-induced pancreatic *β*-cell dysfunction and metabolic syndrome [11].

5′ AMP-activated protein kinase (AMPK) coordinates many different signaling pathways involved in maintenance of energy homeostasis. AMPK is the switch controlling activation of anabolic and catabolic pathways [12]. AMPK regulates lipid metabolism by inactivating acetyl-CoA carboxylase (ACC) and 3-hydroxy-3-methylglutaryl-CoA reductase, resulting in increased lipid oxidation and inhibited cholesterol synthesis [13]. Thus, AMPK activation in the liver suppresses gluconeogenesis, lipogenesis, and cholesterol synthesis. Based upon its function, many studies have suggested that AMPK might be a therapeutic target for treatments for patients with insulin resistance and type II diabetes mellitus [12, 14, 15].

*Rhodiola crenulata* has been used in the traditional Chinese medicine system as a treatment for high altitude illness and diabetes, as well as in tonics [16, 17]. *R. crenulata* root extract (RCE) was recently shown to attenuate abnormal lipid metabolism and improve insulin sensitivity in a rodent model of diabetes [18]. In a previous study [19], we showed that RCE suppressed hepatic gluconeogenesis *via* activation of AMPK signaling. Our results also imply that RCE might suppress hepatic lipogenesis by inactivating key enzymes involved in lipogenesis, including fatty acid synthase (FAS) and ACC [20]. Although these results indicate that RCE is able to ameliorate abnormal glucose and lipid metabolism, the underlying mechanism of RCE requires further elucidation. The goal of the current study is to examine the effects of RCE on hepatic glycogen and lipid metabolism in human HepG2 cells and Sprague–Dawley (SD) rats, as well as to investigate the mechanisms underlying such effects.

## Methods

### Preparation and quantification of RCE

Preparation and analysis of RCE were described in a previous study by our group [19]. Briefly, *Rhodiola crenulata* (Hook. f. & Thomson) H. Ohba roots were obtained from Chuang Song Zong Pharmaceutical Co., Ltd (Kaohsiung,Taiwan). A voucher specimen (NDMCP no.1000901) was deposited in the National Defense Medical Center. The identity of *R. crenulata* was confirmed using the Plant List database. The drug-extract ratio of RCE was 6.25:1. HPLC showed that the RCE contained 3.5 % salidroside.

### Cell culture and animal experiments

Human HepG2 cells [21] were purchased from the American Type Culture Collection (ATCC, Manassas, VA, USA) and maintained in DMEM medium (Gibco Laboratories, Grand Island, NY, USA). Male SD rats were employed in this study as described in a previous study by our group [19]. Briefly, cells were treated with different concentrations of RCE under high glucose conditions (33 mM) for 16 h. SD rats were fed RCE (50 mg/kg, suspended in 1 mL saline) or 1 mL saline by gavage once daily for 3 days, after which the animals were sacrificed and livers were collected. All of these procedures were proven by the Institutional Animal Care and Use Committee of the National Defense Medical Center (IACUC-11-055).

### Glycogen synthesis assay

The glycogen synthesis assay was performed as described in a previous study [22]. Briefly, the assay was carried out with 5.5 mM glucose (2 μCi/mL, [^14^C]-d-glucose) for 30 min at 37 °C, followed by lysis with 0.5 N NaOH and measurement of the radiation intensity of glycogen *via* scintillation counting (Packard).

### Lipogenesis assay

The lipogenesis assay in HepG2 cells was performed using a commercial kit (Cayman Chemical Co.) [23]. Briefly, cells were cultured in 24-well plates, after which 75 *μ*L of Oil Red O solution was added to each well for 15 min. Subsequently, dye extraction solution was added to each well for 20 min. Finally, the absorbance of the solution in each well was measured at 490 nm using an ELISA microplate reader (Spectra Max 190, Molecular Devices).

### Quantitative analysis of lipid metabolic gene expression

Relative gene expression levels of lipogenic enzymes were measured by quantitative real-time PCR (Q-PCR) as described previously [19]. The PCR primers are listed in Table 1.

**Table 1:** Oligonucleotide sequences used in this study

HepG2 cells	Nucleotide sequences (5′-3′)	Reference
SREBP1c sense	TCAGCGAGGCGGCTTTGGAGCAG	[35] (Suh HN, 2008)
SREBP1c antisense	CATGTCTTCGATGTCGGTCAG	
FAS sense	CGGTACGCGACGGCTGCCTG	
FAS antisense	GCTGCTCCACGAACTCAAACACCG	
GAPDH sense	TGGTATCGTGGAAGGACTCA	[36]
GAPDH antisense	AGTGGGTGTCGCTGTTGAAG	
Rat liver		
SREBP1c sense SREBP1c antisense FAS sense	AGGACCCAAGGTGACACCTG GCCGGACGGGTACATCTTT AACTGAACGGCATTACTCGGTC	
FAS antisense	GTGTCCCATGTTGGATTTGGT	
GAPDH sense	AACGGCACAGTCAAGGCTGA	[37]
GAPDH antisense	ACGCCAGTAGACTCCACGACAT	

### Western blotting

Western blotting was performed as described in a previous study by our group [24]. Briefly, immunoblotting was performed using antibodies against AKT, phosphorylated AKT (Thr308 and Ser473, Santa Cruz, CA), CCAAT/enhancer-binding protein (C/EBP), AMPK, phosphorylated AMPK (T172), ACC, phosphorylated ACC (Cell Signaling Tech.), GSK3β, phosphorylated GSK3β (Abcam, Cambridge, MA), GS, phosphorylated GS (Ser641), β-actin (Chemicon, Temecula, CA, USA), and sterol regulatory element-binding protein 1c (SREBP-1c) (Gene Tex, Irvine, CA).

### Statistical analysis

All results are presented as mean ± SEM. Significant differences between group means were determined using one-way ANOVA followed by Bonferroni’s post-hoc test. Analyses were performed using IBM SPSS Statistics version 22 (IBM® SPSS® Statistics 22). Differences were considered to be statistically significant when p-values were less than 0.05.

## Results

### RCE significantly increases AMPK and ACC phosphorylation

**Fig. 1:**
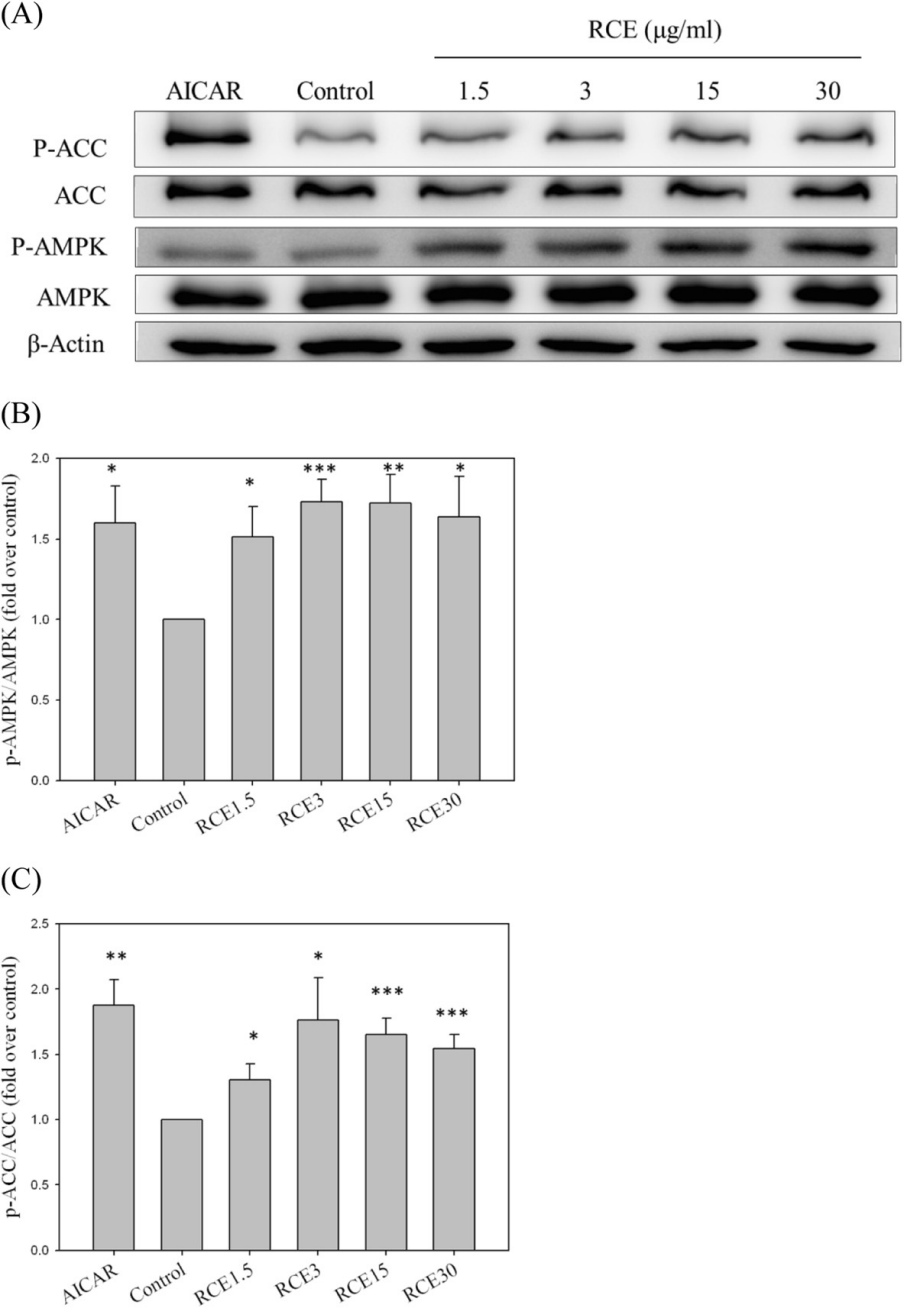
Effect of RCE on phosphorylation of AMPK and ACC in HepG2 cells.HepG2 cells were incubated in high-glucose medium (33 mM) for 16 h prior to the addition of the indicated concentrations of RCE or AICAR (2 mM). After pretreatment with RCE (μg/mL) for 6 h or AICAR for 2 h, p-AMPK and p-ACC expression levels were analyzed by western blotting (**a**). A quantitative analysis of the relative levels of p-AMPK (**b**) and p-ACC (**c**) was conducted. The results represent the mean ± SEM (*n* = 3), ∗*p* < 0.05, ∗∗*p* < 0.01, and ∗∗∗*p* < 0.001 vs. the sample without RCE

To clarify the regulatory effect of RCE on AMPK signaling in the liver, HepG2 cells were incubated with RCE. RCE significantly increased AMPK phosphorylation at T172 (1.51 ± 0.19, 1.73 ± 0.14, 1.73 ± 0.17, and 1.64 ± 0.25-fold over the control sample for 1.5, 3.0, 15.0, and 30.0 *μ*g/mL, respectively; *p* < 0.05, *p* < 0.001, *p* < 0.01, and *p* < 0.05, respectively; Fig. 1a and b). ACC, a major substrate of AMPK, is involved in modulating hepatic lipogenesis, which suppresses conversion of acetyl-CoA to malonyl-CoA and subsequently leads to decreased fatty acid synthesis [25]. ACC phosphorylation following RCE treatment was concentration-dependent (1.31 ± 0.12, 1.76 ± 0.32, 1.65 ± 0.12, and 1.54 ± 0.11-fold over the control sample for 1.5, 3.0, 15.0, and 30.0 *μ*g/mL, respectively; *p* < 0.05, *p* < 0.05, *p* < 0.001, and *p* < 0.001, respectively; Fig. 1a and c). These results indicate that pretreatment with RCE significantly increased the activity of the AMPK-ACC signaling pathway in HepG2 Cells without cytotoxicity under the chosen experimental conditions [19].

### RCE enhances de novo glycogen synthesis and expression of regulatory enzymes

**Fig. 2:**
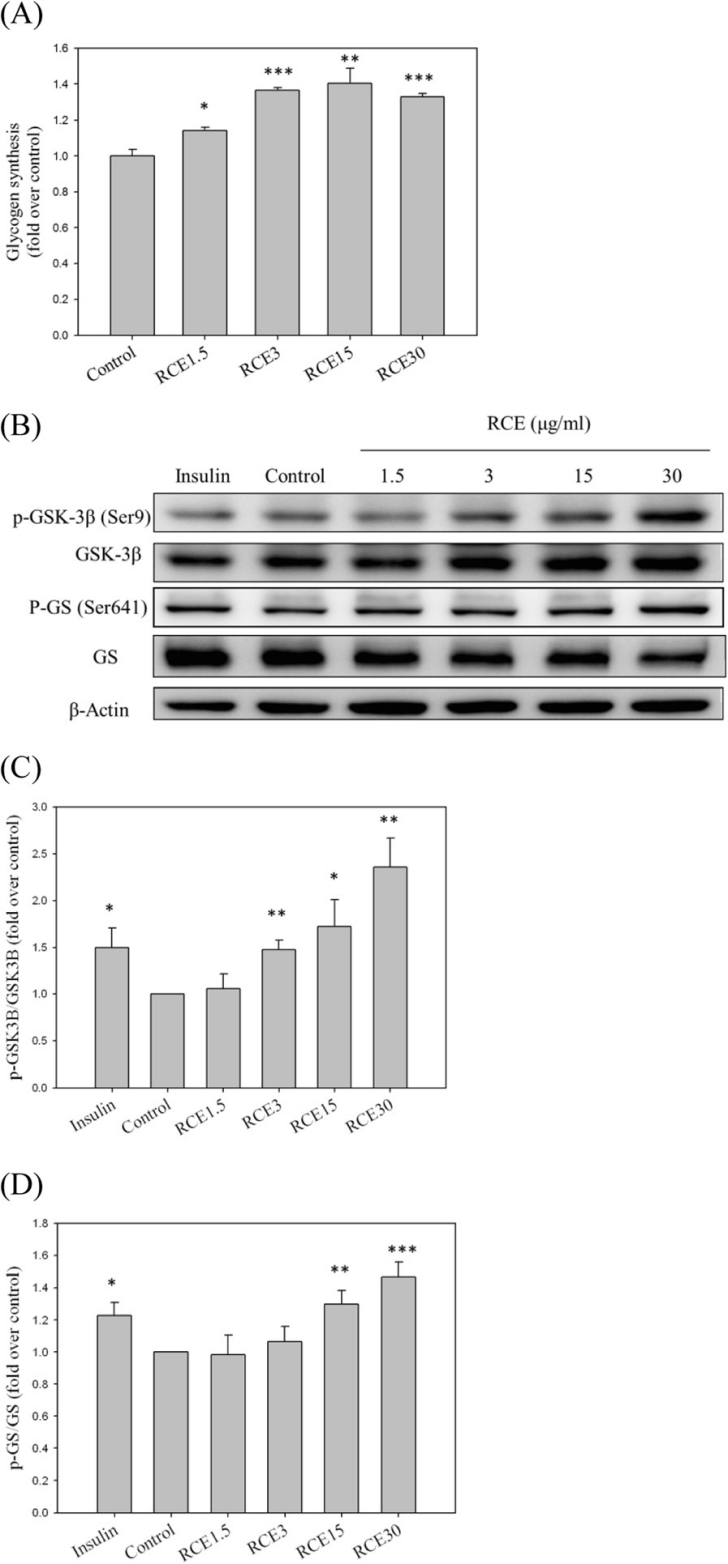
Effects of RCE on glycogen synthesis in HepG2 cells. HepG2 cells were cultured in 24-well plates and exposed to medium without serum for 24 h. The cells were then incubated with RCE (μg/mL) for 6 h or insulin (Ins, 100 nM) for 1 h before ^14^C (U)-d-glucose was added. The intensity of ^14^C in glycogen was analyzed (**a**). Expression levels of p-GSK3ß and p-GS were analyzed by western blotting (**b**). A quantitative analysis of the relative levels of p-GSK3ß (**c**) and p-GS (**d**) was conducted. The results represent the mean ± SEM (*n* = 3), ∗*p* < 0.05, ∗∗*p* < 0.01, ∗∗∗*p* < 0.001 vs. the sample without RCE

To investigate the impact of RCE on glycogen synthesis, HepG2 cells were preincubated with RCE for 6 h. As shown in Fig. 2a, RCE at concentrations of 1.5, 3.0, 15.0, and 30.0 *μ*g/mL significantly increased hepatic glycogen synthesis under high glucose conditions (1.14 ± 0.02, 1.37 ± 0.02, 1.40 ± 0.08, and 1.33 ± 0.02-fold over the control sample, respectively; *p* < 0.05, *p* < 0.001, *p* < 0.01, and *p* < 0.001, respectively, Fig. 2a). In addition, RCE at concentrations of 1.5, 3.0, 15.0, and 30.0 *μ*g/mL significantly enhanced the expression of p*-*GSK3β (1.06 ± 0.15, 1.48 ± 0.10, 1.72 ± 0.29, 2.36 ± 0.31-fold over the control sample, respectively; NS, *p* < 0.01, *p* < 0.05, and *p* < 0.01, respectively; Fig. 2b and c, respectively) and p- GS (0.98 ± 0.12, 1.06 ± 0.09, 1.30 ± 0.08, and 1.47 ± 0.09-fold over the control sample, respectively; NS, NS, *p* < 0.01, and *p* < 0.001, respectively; Fig. 2b and d, respectively). These results indicate that RCE increased glycogen synthesis and expression of regulatory enzymes in HepG2 cells under high glucose conditions.

### RCE reduces lipogenesis and related gene expression

**Fig. 3:**
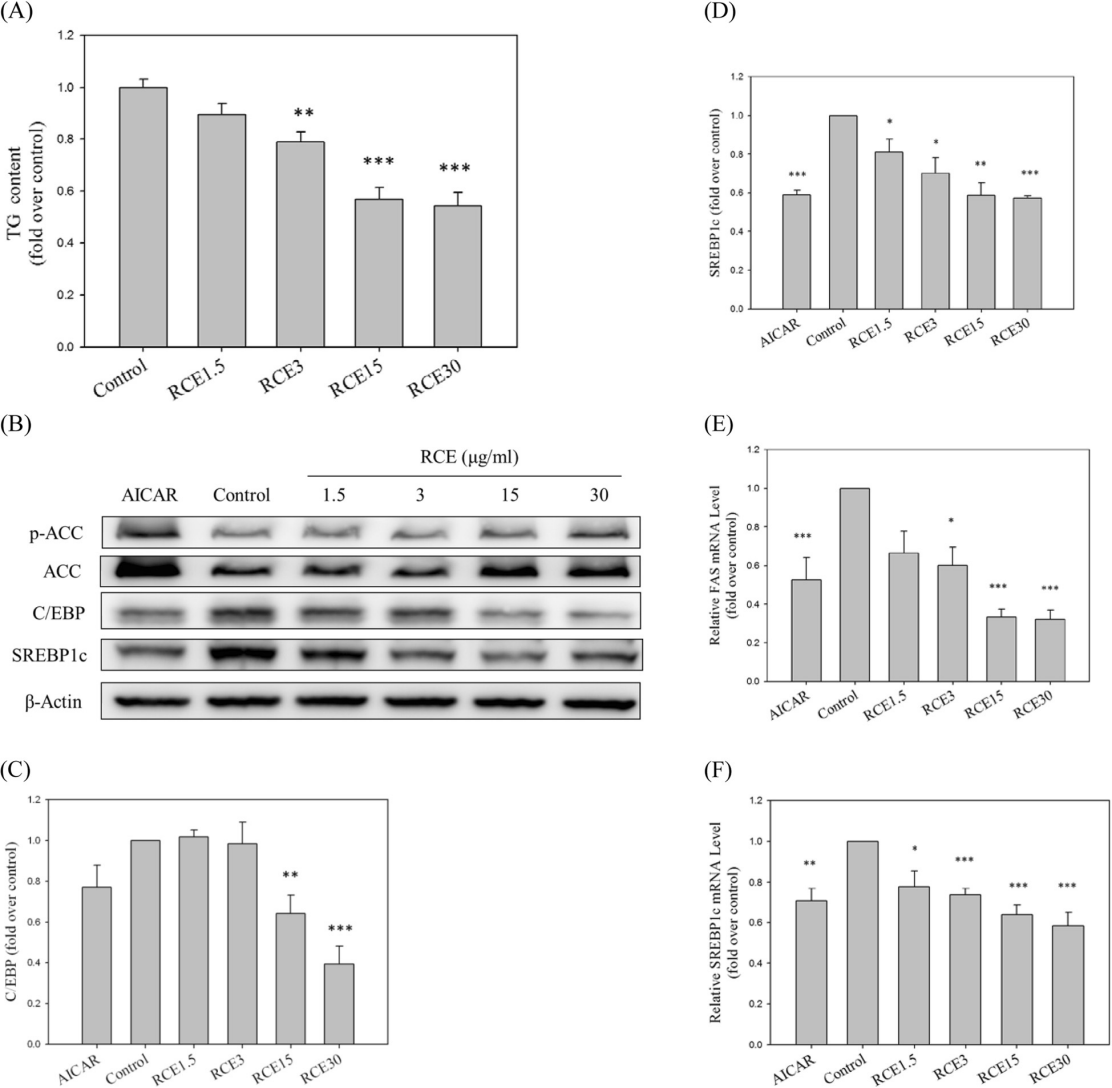
Effects of RCE on TG content and expression of lipogenic enzymes in HepG2 cells. HepG2 cells were cultured in high-glucose (33 mM) medium containing Ins (100 nM) with or without the indicated concentration of RCE (μg/mL) or AICAR (2 mM). Lipogenesis (**a**), relative protein levels SREBP1and C/EBP (**b**), and gene expression (**e** and **f**) were measured. A quantitative analysis of the protein levels of SREBP1 (**c**) and C/EBP (**d**) was conducted. The results represent the mean ± SEM (*n* = 3), ∗*p* < 0.05, ∗∗*p* < 0.01, ∗∗∗*p* < 0.001 vs. the sample without RCE

In addition to the impact of RCE on hepatic glycogen synthesis, the effect of RCE treatment on hepatic lipid metabolism was also examined. RCE at concentrations of 1.5, 3.0, 15.0, and 30.0 μg/mL significantly suppressed hepatic lipogenesis (0.90 ± 0.04, 0.79 ± 0.04, 0.57 ± 0.05, and 0.54 ± 0.05-fold, respectively; NS, *p* < 0.01, *p* < 0.001, and *p* < 0.001, respectively, compared to that of the control group; Fig. 3a). Consistently, we also observed that protein levels of C/EBP (1.02 ± 0.03, 0.98 ± 0.11, 0.64 ± 0.09, and 0.39 ± 0.09-fold for 1.5, 3.0, 15.0, and 30.0 μg/mL, respectively; NS, NS, *p* < 0.01, and *p* < 0.001, respectively, compared to that of the control group; Fig. 3b and c) and SREBP-1c (0.81 ± 0.07, 0.70 ± 0.08, 0.59 ± 0.07, and 0.57 ± 0.01-fold for 1.5, 3.0, 15.0 and 30.0 μg/mL, respectively; *p* < 0.05, *p* < 0.05, *p* < 0.01, and *p* < 0.001, respectively, compared to that of the control group; Fig. 3b and d) were significantly suppressed. Furthermore, RCE treatment also suppressed mRNA levels of lipogenesis-associated, including *FAS*, the key enzyme involved in fatty acid synthesis (0.67 ± 0.11, 0.60 ± 0.09, 0.33 ± 0.04, and 0.32 ± 0.05-fold for 1.5, 3.0, 15.0, and 30.0 *μ*g/mL, respectively; NS, *p* < 0.05, *p* < 0.001, and *p* < 0.001, respectively, compared to that of the control group, Fig. 3e), and *SREBP1c* (0.78 ± 0.08, 0.74 ± 0.03, 0.64 ± 0.05, and 0.58 ± 0.07-fold for 1.5, 3.0, 15.0, and 30.0 μg/mL, respectively; *p* < 0.05, *p* < 0.001, *p* < 0.001, and *p* < 0.001, respectively, compared to that of the control group; Fig. 3f). These results indicate that RCE suppressed fat accumulation in hepatic cells under high glucose conditions. As a positive control, AICAR reduced protein levels of C/EBP (0.77 ± 0.11-fold over the control sample; *NS*; Fig. 3b and c) and SREBP (0.59 ± 0.02-fold over the control sample; *p* < 0.01; Fig. 3b and d), as well as gene expression of *FAS* (0.53 ± 0.12-fold over the control sample; *p* < 0.01; Fig. 3d) and *SREBP1c* (0.71 ± 0.06-fold over the control sample; *p* < 0.01; Fig. 3e).

### Compound C abolishes the effects of RCE on hepatic glycogenesis and lipogenesis

**Fig. 4:**
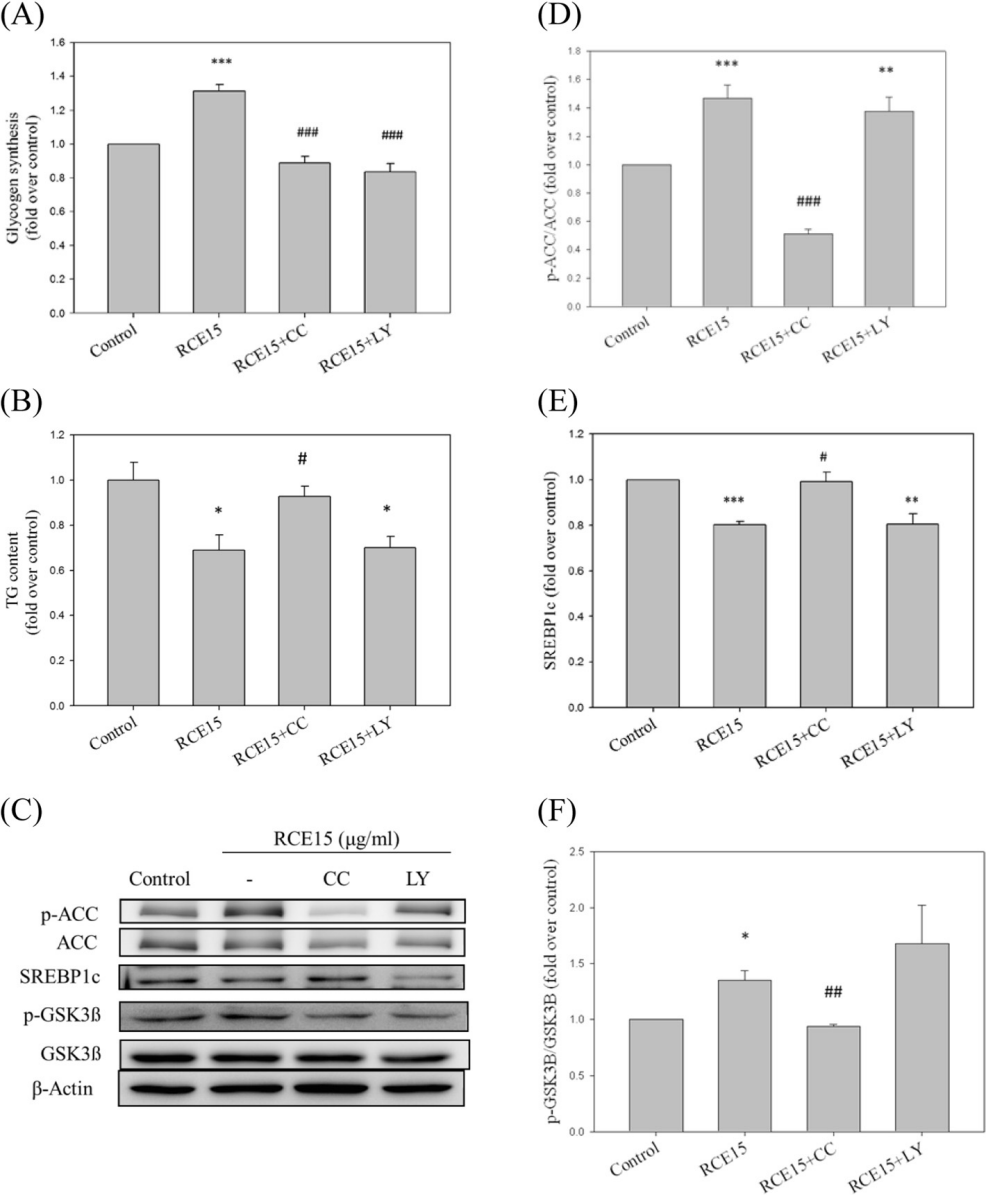
Effects of LY294002 (LY) and compound C (CC) on glycogen synthesis and TG content in HepG2 cells. HepG2 cells were incubated in high-glucose medium (33 mM) for 16 h and then pretreated with RCE for 12 h or kinase inhibitors (20 μM LY294002 or 10 μM compound C) prior to RCE treatment (15 μg/mL) for 1 h. Glycogen synthesis (**a**) and TG content (**b**) were measured. Expression levels of p-ACC, SREBP1c, and p-GSK3ß were measured by western blotting (**c**). A quantitative analysis of the protein levels of p-ACC (**d**), SREBP1c (**e**), and p-GSK3ß (**f**) was conducted. The results represent the mean ± SEM (*n* = 3). ∗*p* < 0.05, ∗∗*p* < 0.01, ∗∗∗*p* < 0.001 vs. the sample without RCE; #*p* < 0.05, ## p < 0.01, and ###*p* < 0.001 vs. the RCE-treated sample

In order to investigate the mechanism underlying the effects of RCE, compound C and LY294002 were employed. RCE-induced glycogen biosynthesis was diminished upon inhibition of signaling by AMPK (from 1.31 ± 0.04 to 0.89 ± 0.04-fold compared with the control sample; *p* < 0.001; Fig. 4a) and PI3K (from 1.31 ± 0.04 to 0.83 ± 0.05-fold compared with the control sample; *p* < 0.001; Fig. 4a). In addition, the inhibitory effect of RCE on lipogenesis was nearly abolished by compound C (from 0.69 ± 0.07 to 0.93 ± 0.05-fold compared with the control sample; *p* < 0.05; Fig. 4b). However, no significant effect of LY294002 treatment was detected (from 0.69 ± 0.07 to 0.70 ± 0.05-fold compared with the control sample; NS; Fig. 4b). Similarly, compound C abolished the effects of RCE on p-ACC (from 1.47 ± 0.09 to 0.51 ± 0.03-fold compared with the control group; *p* < 0.001; Fig. 4c and d), SREBP1c (from 0.80 ± 0.01 to 0.99 ± 0.04-fold compared with the control group; *p* < 0.05; Fig. 4c and e), and p-GSK3ß (from 1.35 ± 0.08 to 0.94 ± 0.02-fold compared with the control group; *p* < 0.01; Fig. 4c and f). This result indicates that the regulatory effects of RCE on hepatic glycogen and lipid metabolism are associated with the AMPK signaling pathway.

### RCE administration suppresses lipogenesis-related gene expression in the rat liver

**Fig. 5:**
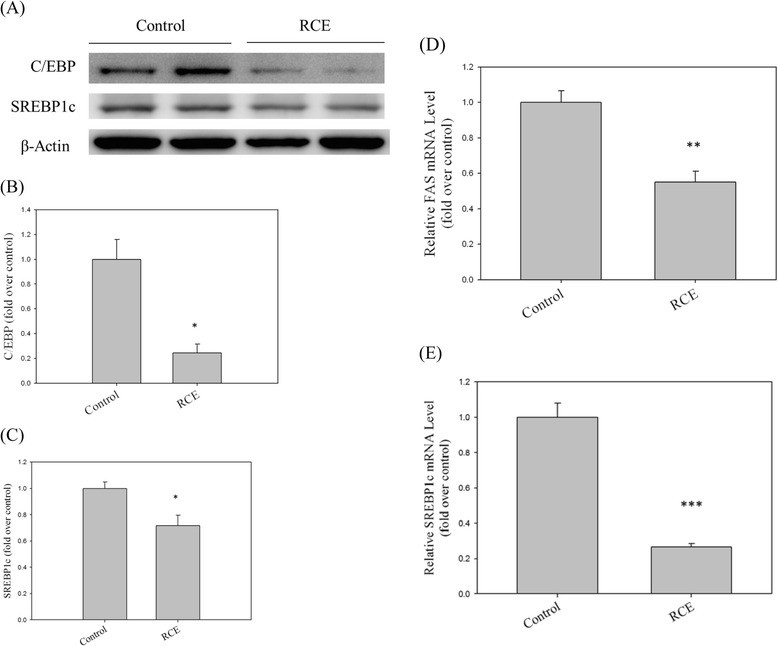
Effects of RCE on lipogenic protein and gene expression in rat liver. Protein (**a**) and gene (**d** and **e**) expression levels of C/EBP and SREBP-1c were analyzed by western blotting and Q-PCR, respectively. A quantitative analysis of the protein levels of C/EBP (**b**) and SREBP-1c (**c**) was conducted. The results represent the mean ± SEM (*n* = 3). ∗*p* < 0.05, ∗∗*p* < 0.01, and ∗∗∗*p* < 0.001 vs. the sample without RCE

To assess the effects of RCE treatment in vivo, a rodent model was employed. Hepatic C/EBP and SREBP1c protein levels (0.24 ± 0.07 and 0.72 ± 0.08-fold compared to the control group; *p* < 0.05, respectively; Fig. 5a, b, and c) were significantly decreased in the RCE-treated animals. Similarly, mRNA levels of lipogenesis-related genes *FAS* and *SREBP1c* in the rat liver were also suppressed by RCE treatment (0.55 ± 0.06 and 0.26 ± 0.02-fold compared to the control group; *p* < 0.01 and *p* < 0.001, respectively; Fig. 5d and e). These results indicate that the in vivo effects of RCE are similar to its in vitro effects*.*

## Discussion

**Fig. 6:**
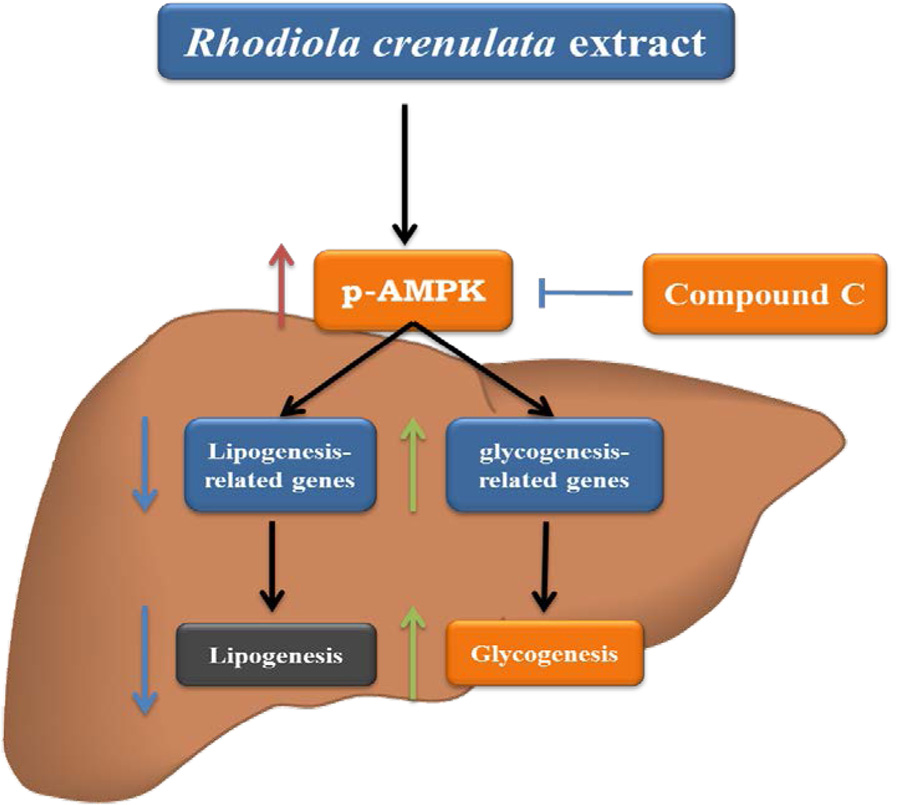
The regulation of RCE on hepatic glycogen and lipid metabolism. RCE regulates hepatic glycogen and lipid metabolism through the AMPK signaling pathway

The liver is an important organ for whole-body energy homeostasis because of its regulation of glucose and lipid metabolism [12]. Hepatic AMPK activation suppresses anabolic metabolism, including cholesterol and fatty acid synthesis, and increases fatty acid oxidation by regulating gene expression and downstream enzymatic activities. Thus, hepatic AMPK activation is commonly regarded as a strategy for managing metabolic syndrome [12] and related symptoms, such as fatty liver. In a previous study, we showed that RCE increased hepatic AMPK activation [19]. In this study, RCE significantly increased AMPK activation and regulated glycogen synthesis and lipogenesis in hepatic cells. The hypolipidemic effect of RCE was verified by the decreased expression levels of genes involved in fatty acid biosynthesis (SREBP-1c, C/EBP, and FAS) and increased fatty acid β-oxidation (phosphorylation of ACC) in HepG2 cells. In addition, RCE inhibited protein and gene expression of lipogenesis-related genes (SREBP-1c, C/EBP, and FAS) in the rat liver. These results suggest that RCE regulates hepatic glycogen and lipid metabolism *via* the AMPK pathway (Fig. 6).

Biosynthesis of glycogen is decreased in hepatocytes under high glucose conditions [26] and patients with insulin resistance [27]. Impaired glucose metabolism is associated with hepatic lipid accumulation. However, most studies have focused on improving abnormal lipid metabolism rather than glycogen synthesis. In this study, we simulated hepatic insulin-resistant conditions by incubating HepG2 cells in 33 mM high-glucose medium. Pretreatment with RCE significantly increased glycogen synthesis under high glucose conditions (Fig. 2a). The effect of RCE is similar to that of thiazolidinedione, a clinical antidiabetic drug that increases hepatic glycogen content in diabetic rodents [28]. In addition, we showed that RCE significantly increased GSK3β phosphorylation at Ser9, thereby inhibiting its activity, which subsequently increased GS activity and glycogen biosynthesis in HepG2 cells (Fig. 2b, c, and d). These results indicate that RCE exerted a beneficial effect on hepatic glycogen metabolism under high glucose conditions. Although the PI3K/AKT signaling pathway is crucial in the regulation of glycogen biosynthesis [7], RCE treatment did not promote phosphorylation of AKT at T308 or S473 in our previous study [19]. These results are thus consistent with the effect of metformin, an AMPK activator, as an inhibitor of Akt phosphorylation under high glucose conditions [29]. Thus, improvement of glycogen synthesis by RCE is not dependent on the PI3K/AKT signaling pathway. In fact, phosphorylation of GSK3ß was also directly regulated by AMPK activation [30]. Based on these results, we suggest that RCE might increase glycogen synthesis *via* the AMPK-GSK3β -GS pathway, despite the fact that the physiological significance of such an effect is still unclear. Furthermore, a previous study mentioned that regulation of GSK3 activity in pancreatic beta cells enhanced cell viability and proliferation, an effect that would benefit patients with type I and II diabetes [31]. The possibility that RCE improves insulin sensitivity partially *via* the AMPK-GSK3 pathway in pancreatic beta cells requires further investigation.

The pathological mechanism of NAFLD is excessive lipid accumulation in the liver due to imbalanced fatty acid synthesis and oxidation [32], which are orchestrated by the interaction of multiple factors, including SREBP-1, an important transcriptional modulator involved in hepatic lipid metabolism that is involved in transcriptional regulation of lipogenic enzymes (FAS and ACC), regulation of fatty acid biosynthesis and VLDL assembly, and intracellular fatty acid trafficking [27, 33]. In addition, overexpression of hepatic SREBP-1 was shown to be associated with hyperlipidemia in rodent models of obesity and type II diabetes [12]. Here, we showed that RCE treatment significantly suppressed in vitro and in vivo SREBP-1c expression. In addition, SREBP1 expression was inhibited by AMPK activation [34]. Thus, we suggest that RCE regulated hepatic lipid accumulation, at least partially by suppression of SREBP-1c expression. Similarly, we found that RCE significantly increased hepatic AMPK activation, while it phosphorylated and inactivated downstream substrates of AMPK, including ACC and FAS. Phosphorylation of ACC suppressed conversion of acetyl-CoA to malonyl-CoA, which contributed to inhibited lipid synthesis, increased mitochondrial fatty acid oxidation, and insulin-sensitization [25]. This result suggests that the insulin-sensitizing effect of RCE might have been a result of activation of the AMPK-ACC signaling pathway. RCE exerted beneficial effects by inhibiting fat accumulation and promoting fat oxidation in the liver, suggesting that it might be a potential preventive agent for NAFLD.

Glycogen metabolism is crucial for long-term physical performance. We demonstrated that RCE improved glycogen metabolism, which implies that RCE could enhance the physical performance of diabetic patients. A recent study indicated that activation of AMPK by an herbal medicine produced beneficial effects in a rodent model of ethanol-induced hepatosteatosis [35]. Metformin, an AMPK activator, alleviated hepatic steatosis in obese mice [36]. With the capacity to activate AMPK, further studies are required to elucidate whether RCE can ameliorate hepatic steatosis in alcoholic patients. In humans, obesity and plasma TG concentration are significantly associated with the TG level in the liver [37]. Wang et al. indicated that RCE decreased the plasma concentration of TG in a rodent model [18]. Lee et al. indicated that RCE exerted antiadipogenic effects involved in endogenous antioxidant enzyme response and proline-mediated pentose phosphate pathway in differentiated adipocytes [38]. We showed that RCE decreased TG content in HepG2 cells. Taken together, these results suggest that RCE might be useful for the management of systemic lipid metabolism.

## Conclusion

In this study, we demonstrated the ability of RCE to regulate glycogen and lipid metabolism in the liver. Our results suggest that *R. crenulata* might be a potential therapeutic agent for NAFLD.

### Ethics approval and consent to participate

The animal experimental procedures were performed according to the Institutional Animal Care and Use Committee of the National Defense Medical Center (IACUC-11-055).

### Consent for publication

Not applicable

### Availability of data and materials

The data and materials have been presented in the main manuscript.

## Abbreviations

RCE: *Rhodiola crenulata* root extract
NAFLD: nonalcoholic fatty liver disease
AMPK: AMP-activated protein kinase
ACC: acetyl-CoA carboxylase
GSK3β: glycogen synthase kinase 3β
GS: glycogen synthase
FAS: fatty acid synthase
C/EBP: CCAAT/enhancer-binding protein
SREBP-1c: sterol regulatory element-binding protein 1c
TG: triglycerides
